# Genetic Susceptible Locus in *NOTCH2* Interacts with Arsenic in Drinking Water on Risk of Type 2 Diabetes

**DOI:** 10.1371/journal.pone.0070792

**Published:** 2013-08-14

**Authors:** Wen-Chi Pan, Molly L. Kile, Wei Jie Seow, Xihong Lin, Quazi Quamruzzaman, Mahmuder Rahman, Golam Mahiuddin, Golam Mostofa, Quan Lu, David C. Christiani

**Affiliations:** 1 Department of Environmental Health, Harvard School of Public Health, Boston, Massachusetts, United States of America; 2 Department of Public Health, College of Public Health and Human Sciences, Oregon State University, Corvallis, Oregon, United States of America; 3 Department of Biostatistics, Harvard School of Public Health, Boston, Massachusetts, United States of America; 4 Department of Environmental Research, Dhaka Community Hospital, Dhaka, Bangladesh; 5 Department of Genetics and Complex Diseases, Harvard School of Public Health, Boston, Massachusetts, United States of America; University of Louisville, United States of America

## Abstract

**Background:**

Chronic exposure to arsenic in drinking water is associated with increased risk of type 2 diabetes mellitus (T2DM) but the underlying molecular mechanism remains unclear.

**Objectives:**

This study evaluated the interaction between single nucleotide polymorphisms (SNPs) in genes associated with diabetes and arsenic exposure in drinking water on the risk of developing T2DM.

**Methods:**

In 2009–2011, we conducted a follow up study of 957 Bangladeshi adults who participated in a case-control study of arsenic-induced skin lesions in 2001–2003. Logistic regression models were used to evaluate the association between 38 SNPs in 18 genes and risk of T2DM measured at follow up. T2DM was defined as having a blood hemoglobin A1C level greater than or equal to 6.5% at follow-up. Arsenic exposure was characterized by drinking water samples collected from participants' tubewells. False discovery rates were applied in the analysis to control for multiple comparisons.

**Results:**

Median arsenic levels in 2001–2003 were higher among diabetic participants compared with non-diabetic ones (71.6 µg/L vs. 12.5 µg/L, p-value <0.001). Three SNPs in *ADAMTS9* were nominally associated with increased risk of T2DM (rs17070905, Odds Ratio (OR)  = 2.30, 95% confidence interval (CI) 1.17–4.50; rs17070967, OR = 2.02, 95%CI 1.00–4.06; rs6766801, OR = 2.33, 95%CI 1.18–4.60), but these associations did not reach the statistical significance after adjusting for multiple comparisons. A significant interaction between arsenic and *NOTCH2* (rs699780) was observed which significantly increased the risk of T2DM (p for interaction = 0.003; q-value = 0.021). Further restricted analysis among participants exposed to water arsenic of less than 148 µg/L showed consistent results for interaction between the NOTCH2 variant and arsenic exposure on T2DM (p for interaction  = 0.048; q-value = 0.004).

**Conclusions:**

These findings suggest that genetic variation in *NOTCH2* increased susceptibility to T2DM among people exposed to inorganic arsenic. Additionally, genetic variants in *ADAMTS9* may increase the risk of T2DM.

## Introduction

The prevalence of diabetes is increasing globally. In 2000, the prevalence of diabetes was 2.8% and is projected to reach 4.4% in 2030 [1]. In the United States, the prevalence of diabetes is estimated to be 8.3% in 2010 [2]. Type 2 diabetes mellitus (T2DM) is a complex disease with multiple contributing factors. Environmental factors such as diet [3], [4] , life style [5], and cigarettes smoking [6] are positively associated with onset of T2DM. Genetic components have also been shown to be associated with T2DM [7]. With its increased prevalence and the enormous number of cases, diabetes is now a major health problem worldwide.

Over 140 million people worldwide are exposed to arsenic-contaminated water with levels over the World Health Organization's recommended guideline value of 10 µg/L [8]. Epidemiologic studies show that chronic exposure to arsenic in drinking water, a toxic environmental pollutant, is associated with significant increased risk of T2DM [9]–[18]. *In vitro* studies that explored the pathogenesis of arsenic-induced T2DM have implicated the involvement of several biological processes, including the protein kinase B (PKB/Akt) pathway [19], [20], calcium-dependent calpain-10 proteolysis of SNAP-25 [21], [22], cellular adaptive response of oxidative stress [23], [24], inhibition of glucose-dependent insulin secretion [25],and endoplasmic reticulum stress [26]. However, the molecular genetic mechanisms underlying arsenic-induced T2DM remain incompletely understood.

Hypothesis-generating approaches are useful tools for discovering novel mechanisms for identifying risk factors for complex diseases like T2DM. For example, genome wide association studies have identified 27 genetic variants that are associated with T2DM [7] that had not been observed in hypothesis-driven studies. This evidence shows that hypothesis-generating approach is an alternative approach to discover novel genetic factors associated with T2DM.

Meta-analysis of T2DM with 141,000 participants has identified 27 loci that are associated with T2DM. It is possible that some of these variants may contribute to arsenic-induced T2DM. In this paper, we examined gene-environment interactions between single nucleotide polymorphisms (SNPs) in susceptible genes of T2DM and arsenic exposure in drinking water on risks of T2DM in a Bangladesh population.

## Materials and Methods

### Study design and participants

This is a follow up study based on a case-control study conducted in 2001–2003 that was designed to identify environmental and genetic risk factors for arsenic-related skin lesions. The original case-control study enrolled 900 individuals with skin lesions and 900 individuals without skin lesions who lived in rural areas of Bangladesh where arsenic contaminated groundwater is commonly used for drinking water [27]. From 2009 to 2011, we successfully re-contacted 1,542 of these participants and 957 individuals agreed to participate in this follow up study. Questionnaires were used to collect information on medical history, cigarette smoking, and other risk factors in both 2001–2003 (baseline) and 2009–2011 (follow-up). At both time periods, water arsenic exposures were measured. Hemoglobin A1C level was only measured at follow-up.

Of the 957 individuals, seventeen did not have adequate genomic DNA for genotyping and nineteen individuals who reported a diagnosis of T2DM prior to 2001 were excluded. Additionally, two individuals who did not provide information regarding their diabetes status in 2009–2011 were also excluded. Thus, 919 individuals were included in this analysis. This study was approved by the Institutional Review Boards of Harvard School of Public Health and Dhaka Community Hospital. All participants provided written informed consent prior to participation in any study activity.

### Arsenic Exposure in Drinking Water

Arsenic exposure assessment in this study has been described previously [27], [28]. Briefly, trained interviewers collected drinking water samples from the tubewell which was identified as the primary source of drinking water for each participant. Water samples were preserved with nitric acid and then analyzed for arsenic by inductively coupled plasma mass spectroscopy by Environmental Laboratory Services North Syracuse, NY, USA following the U.S. Environmental Protection Agency Method 200.8 (U.S. Environmental Protection Agency 1994). The limit of detection was 1 μg As/L. Water samples below the limit of detection were recorded as 0.5 μg/L.

### Identification of Type 2 Diabetes Cases

Two milliliters of blood was collected from all participants at follow-up by venipuncture into Fluoride/EDTA vacutainers. These samples were analyzed for hemoglobin A1C within 24 hours by the Diabetes Center in Pabna, Bangladesh using the NycoCard HbA1C system following the manufacturer's instructions (NycoCard HbA1C, Axis-Shield, Norway). Individuals were defined as having T2DM if hemoglobin A1C ≥6.5% following the recommendation of the International Expert Committee on Diabetes [29], [30].

### Selection Criterion of Single Nucleotide Polymorphism

Thirty-eight (SNPs) with potential biological functions were identified by searching the HapMap database (http://hapmap.ncbi.nlm.nih.gov/) using the following criteria:1) SNPs must be located in the 32 reported susceptible genes of T2DM discovered by meta-analysis of the exiting genome-wide association study [7]; 2) SNPs were selected based on the population of Gujarati Indians in Houston; 3) SNPs must be located in the 5′-Untranslated Region (5′-UTR), 3′-Untranslated Region (3′-UTR), or non-synonymous; 4) the minor allele frequency must be equal or greater than 5% in the Gujarati Indians in Houston population. All SNPs included in the analysis have potential biological function. Non-synonymous SNPs result in the change of amino acid sequence that may influence tertiary structure of target proteins. 5′-UTR SNPs may affect the translational efficiency through modulating the mRNA stability. 3′-UTR SNPs may change the mRNA stability because micro RNA can potentially bind to this region and further affect the half-life of pre-mRNA.

### Genotyping Procedure of Single Nucleotide Polymorphism

Briefly, 30 μL of DNA was aliquotted into the 96 wells plates with concentrations ranging from 5 to 20 ng/μL. Genotyping was performed on 5–20 ng/µL of DNA using Sequenome MassARRAY iPLEX (San Diego, CA) by the Partners HealthCare Center for Personalized Genetic Medicine (Boston, MA.). SNPs that failed in test of Hardy-Weinberg Equilibrium with p-values <0.001 were excluded from the analysis. Ten percent of the samples were randomly selected and analyzed in duplicate for quality control, and rates of genotyping concordance were greater than 99%.

### Statistical Analysis

Wilcoxon rank sum test with continuity correction for continuous variables and Fisher's exact test for categorized variables were used to compare baseline characteristics between individuals with and without T2DM. The odds ratio (OR), 95% confidence interval (CI) and p-value were estimated by multiple logistic regression. Additive genetic models assessed the association between SNPs and risk of T2DM, as well as the interaction between SNPs and arsenic in drinking water at baseline (2001–2003) on the risk of T2DM. The covariates in the analyses were based on the information obtained in 2001–2003 including age (continuous), sex, body mass index (continuous), smoking status (never, and past or current), status of skin lesion (present, absent), and natural logarithm transformed water arsenic. Education and marital status were not included in the final model because it did not significantly change the point estimates.

Examination of the data shows nonlinear arsenic exposure effect on T2DM. Hence, we modeled arsenic effects nonlinearly using penalized splines when studying the effects of individual SNPs and arsenic exposure on T2DM, adjusting for age, sex, body mass index (BMI) (calculated as weight (kg)/height (m)^2^), skin lesion, and smoking. We also performed gene-level analyses by applying kernel machine regression [31] to assess the joint effects of the SNPs in a gene on T2DM risk, where log arsenic was modeled using polynomial regression methods with 3 degrees of freedom to model its nonlinear effect.

We next studied the interactions between a SNP and arsenic on T2DM risk. In view of the nonlinear arsenic effect, we modeled log arsenic using piecewise linear regression with knots at 1, 3, 5 and calculate the cross-product terms of SNPs. Using the fitted interaction model, we estimated the effect of arsenic on T2DM for each SNP. The p-values for the interaction terms were calculated using the likelihood ratio test.

The adjustments of multiple comparisons were controlled by using false discovery rates (FDR) methods [32] that controls the probability of false discovery at a level set by researchers. In this study, we set the FDR that is less or equal than 5% (q-value less or equal to 0.05) to minimize false positive findings. Thus, any two-sided p-values <0.05 were considered statistically significant. Additional information regarding raw data, scatter plots, and analyses on continuous manner of glycated hemoglobin A1C can be found in supplementary (Table S1, S2, S3, and S4; Figure S1, S2, and S3). All statistical analyses were performed in R version 2.13 [33].

## Results

In total, 919 individuals who had adequate DNA for genotyping and did not report having T2DM at baseline (2001–2003) were included in this analysis. Based on levels of blood hemoglobin A1C in 2009–2011, we identified 83 cases of T2DM yielding a crude prevalence of 9.0%. Table 1 shows the comparison of baseline characteristics among participants with and without T2DM. Overall, participants with T2DM had higher BMI, were older, and had greater exposure to arsenic contaminated drinking water compared with participants without T2DM. The median levels of arsenic in drinking water in participants with T2DM was 71.6 μg/L compared to 12.5 μg/L for participants without T2DM (p-value <0·001). Male participants with T2DM were also more likely to be ever or current smokers (42.1% versus 28.1%, P = 0.004). The frequency of skin lesion status did not significantly differ between participants with and without T2DM (57.4% versus 51.8%, p-value = 0.35).

**Table 1:** Distribution of Demographic, Lifestyle, and Arsenic Exposure Variables by Diabetes Status (n = 919) in Dhaka, Bangladesh, 2001–2003 (Baseline).

		Diabetes [n(%)]		
Variable^a^		No (n = 836)	Yes (n = 83)	p-value^b^
Age, years	Mean (SD)	33.46 (11.7)	39.8 (10.8)	<0.001
	Median (IQR)	33.0 (18.0)	40.0 (14.0)	
Sex	Male	505 (60.4)	53 (63.9)	0.558
	Female	331 (39.6)	30 (36.1)	
Marital status	Single	222 (26.6)	9 (10.8)	0.003
	Married	598 (71.5)	71 (85.5)	
	Widowed or divorced	16 (1.9)	3 (3.6)	
Education attainment, year	0–6	487 (58.3)	50 (60.2)	0.888
	7–12	319 (38.2)	30 (36.1)	
	>12	29 (3.5)	3 (3.6)	
BMI, kg/m^2^	<24.9	728 (93.5)	59 (71.1)	<0.001
	≧25.0	54 (6.4)	24 (28.9)	
Cigarette smoking (among male only)	Never	599 (71.9)	48 (57.8)	0.004
	Past or current	234 (28.1)	35 (42.1)	
Skin lesion	Yes	480 (57.4)	43 (51.8)	0.353
	No	356 (42.6)	40 (48.2)	
Arsenic in drinking water in baseline (2001–2003), μg/L	Mean (SD)	141.9 (249.6)	207.2 (279.0)	<0.001
	Median (IQR)	12.5 (125.5)	71.6 (269.8)	

a SD and IQR denoted standard deviation and interquartile range, respectively.

b P-values from Fisher's exact test for sex, marital status, education attainment, BMI, cigarette smoking, and skin lesion; or from Wilcoxon rank sum test with continuity correction for age, and arsenic in drinking water. 1 participant was missing for education and 8 participants were missing for smoking status.

Table S1 showed the summary characteristics of the SNPs genotyped in this population. The minor allele frequency of the 38 SNPs ranged from 5% to 45% indicating that these are common variants in a Bangladeshi population. Of these SNPs, 9 SNPs are non-synonymous, 2 SNPs occur in 5′-UTR, and 27 in 3′-UTR.

We observed that three SNPs (rs17070905, rs17070967, and rs6768801), all located within the *ADAMTS9* gene, were associated with an increased risk of T2DM (Table 2). After adjusting for age, sex, BMI, smoking status, skin lesion, and arsenic in drinking water at baseline (2001–2003), the adjusted ORs were 2.30 (95% CI: 1.17–4.50, p-value = 0.015), 2.02 (95% CI: 1.00–4.06, p-value = 0.049), and 2.33 (95% CI: 1.18–4.60, p-value = 0.015) for rs17070905, rs17070967, and rs6766801 respectively. While the nominal p-values for these 3 SNPs were significant (p<0.05), they were no longer significant after adjusting for multiple comparisons using FDR (q-value > 0.05 for all SNPs). Furthermore, the overall association of *ADAMTS9* and risks of T2DM reached statistical significance using kernel machine based multi-marker test (p-value  = 0.006), but was not statistically significant after adjusting for multiple comparisons at the FDR 5% level (q-value = 0.066).

**Table 2:** Associations between SNPs and risks of type 2 diabetes in Dhaka, Bangladesh, 2001–2011.

Marker	Gene	Adjusted Odds Ratio^a^	95% Confidence Interval	p-value^b^	q-value
rs17070905	ADAMTS9	2.30	1.17–4.50	0.015	0.247
rs17070967	ADAMTS9	2.02	1.00–4.06	0.049	0.408
rs6766801	ADAMTS9	2.33	1.18–4.60	0.015	0.247

a Models were adjusted for age, sex, BMI, smoking, skin lesion, and arsenic in drinking water using penalized splines.

b P-value for overall effects of three markers in *ADAMST9* was 0.006 (q-value = 0.066) using kernel machine regression adjusting for age, sex, BMI, smoking, skin lesion, and arsenic in drinking water.

In order to assess the interaction between SNPs and water arsenic on risk of T2DM, for each SNP, we modeled the effect of arsenic exposure using piecewise linear regression in the interaction model. In Table 3, rs1051055 (*CDC123*), rs699780 (*NOTCH2*), and rs2688 (*TCF2*) interacted with arsenic in drinking water to significantly increase the risk of T2DM with p-values for the SNP-arsenic interactions as 0.008, 0.003, and 0.003, respectively. These gene-environment interactions remained significant after controlling for multiple comparisons (rs1051055, q-value  = 0.033; rs699780, q-value = 0.021; rs2688, q-value = 0.021).

**Table 3:** Interaction between SNPs and arsenic in drinking water on risk of type 2 diabetes in Dhaka, Bangladesh, 2001–2011.

Marker	Gene	p-value for interaction^a^	q-value^b^
rs1051055	CDC123	0.008^c^	0.033
rs699780	NOTCH2	0.003^c^	0.021
rs2688	TCF2	0.003^c^	0.021

a Models were adjusted for age, sex, BMI, smoking, skin lesion, SNPs and arsenic in drinking water using piece-wise regression models.

b Q-values were calculated using FDR method for p for interaction among whole population.

c P for interactions were 0.613 for rs1051055 (*CDC123*), 0.048 for rs699780 (*NOTCH2*), and 0.219 for rs2688 (*TCF2*) among people exposed to water arsenic less than 148 μg/L.

To investigate the robustness of interaction between SNPs and water arsenic exposure, we repeated these models in a subset of this population that was exposed to water arsenic below 148 μg/L (the 75^th^ of arsenic exposure from drinking water). In these restricted analyses, only the interaction term for rs699780 (*NOTCH2*) remained statistical significance (p = 0.048). The interaction terms for rs1051055 (p = 0.613) and rs2688 (p = 0.219) were not significant. Therefore, we only considered rs699780 in *NOTCH2* for further analyses.

**Figure 1:**
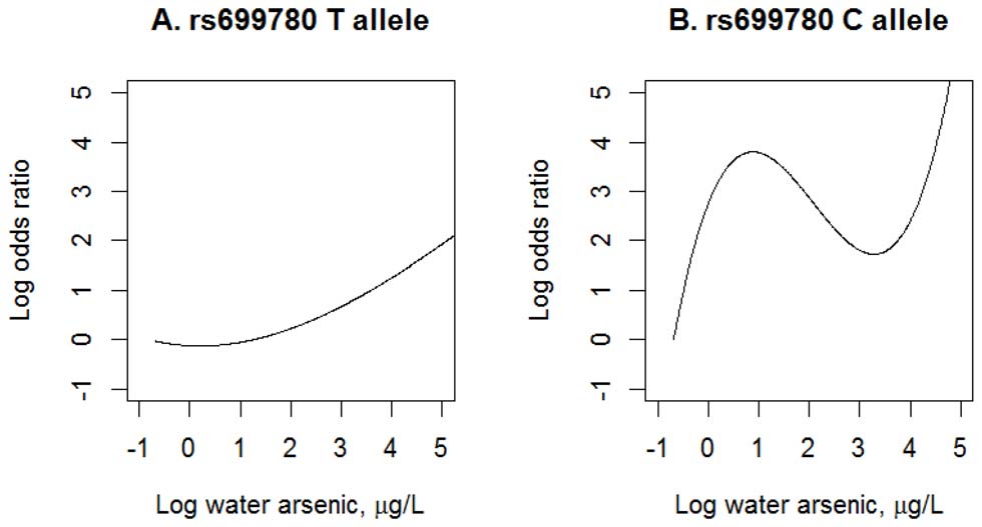
Exposure-response relationship between arsenic in drinking water and diabetes by *NOTCH2* in Dhaka, Bangladesh, 2001–2011. Restricted analyses were performed among people exposed to water arsenic below 148 μg/L adjusting for age, sex, BMI, smoking, skin lesion, and arsenic in drinking water using cubic polynomial models. P for interaction was 0.012. Sample sizes were 316 for TT carriers and 325 for TC/CC carriers of rs699780. The solid line indicated the estimated odds ratio using lowest exposure levels of water arsenic as the reference. Water arsenic was natural logarithm- transformed.

To further understand how the rs699780 (*NOTCH2*) modified the concentration-response relationship between arsenic exposure and T2DM, we examined the nonlinear arsenic effects using polynomial regression with 3 degrees of freedom and stratified data into the two allele groups (TT vs. CT/CC) for rs699780 (*NOTCH2*), where C alleles were characterized as the minor allele and T alleles were characterized as the major allele in this population. Participants with at least one copy of C allele in rs699780 (Figure 1B) had significantly higher risk of T2DM at any given levels of arsenic exposure from drinking water (p for interaction  = 0.012) compared to participants without C allele (Figure 1A). Additionally, the relationship between arsenic exposure and T2DM among participants with at least a C allele showed a non-monotonic increasing trend, whereas those who had T allele had a simple linear relationship.

## Discussion

Using a candidate gene approach, this study observed that SNPs within *NOTCH2* gene significantly increased susceptibility to arsenic-induced T2DM where individuals who carried the C allele were significantly more likely to be diagnosed with T2DM at follow up compared to individuals with the T allele for any given arsenic exposure. In addition, we found that one SNP (rs699780) located in *NOTCH2* with potential biological function significantly interacted with arsenic in drinking water on the risks of T2DM. Restricting the analysis among participants exposed to arsenic concentration in the drinking water at lower levels (<148 μg/L) showed that the interaction between this SNP and arsenic exposure in the drinking water remained statistically significant, suggesting that the interaction effects was not driven by highly exposed individuals. Furthermore, the risk of T2DM was significantly higher in individuals who carried C allele of rs699780 which indicated that the genetic variant in *NOTCH2* increased susceptibility to T2DM among people exposed to arsenic. Furthermore, we identified 3 SNPs in the *ADAMTS9* gene that were nominally associated with the risks of T2DM in an arsenic-exposed population in Bangladesh. These findings provided additional information that*ADAMTS9*, the T2DM susceptible gene discovered in the Caucasian population [7], may play a role in etiology of T2DM in the Bangladesh population.

The observation that genetic variants in *NOTCH2* interact with arsenic exposure to increase the risk of T2DM provides new insights into the pathogenesis of arsenic-related diabetes. It is known that chronic exposure to arsenic in drinking water is associated with an increase of interleukin-6 (IL-6) expression in circulating lymphocytes [34]. Additionally, animal evidence shows that chronic exposure to arsenite decreases toll-like receptor (TLR) signaling which controls the expression of tumor necrosis factor alpha , a cytokine strongly associated with risks of T2DM [35]. While *NOTCH2* is generally involved in development and cell fate decision, there is increasing evidence that *NOTCH2* signaling is important also for immune functions [36]. In particular, NOTCH2 signaling pathway has been shown to suppress the TLR-induced IL-6 and tumor necrosis factor alphaexpression in macrophages [37]. Taken together, *NOTCH2* and arsenic exposure may act through TLR signaling that regulates the innate immune response to increase the risk of T2DM. As such, genetic variants within *NOTCH2* would modify the arsenic-associated diabetic risks.

The prevalence of diabetes in our study was 9% (Table 1) which was close to the value in the U.S. (8.3%) in 2010 [2]. However, the prevalence of obesity (BMI greater than 30.0) was merely 1.0% (9 out of 919 participants) in our follow up study compared to the obesity status in U.S. where 35.7% of adults have a BMI greater than 30 in 2009–2010 [38]. This pattern suggests that other environmental factors may significantly contribute the unexpected high prevalence of diabetes in our study. One of the possible explanations may be the much higher arsenic exposure from drinking water compared to the values in U.S. The median arsenic exposure in our study is 15.1 μg/L whereas the value is only around 2 μg/L in U.S. [39]. This evidence may indicate that beyond the personal health status (e.g. BMI), environmental factors such as arsenic exposure should be considered as an important risk factors for diabetes in Bangladesh.

This study had several limitations that should be considered. First, this is a novel finding that needs to be replicated in other populations to confirm the significant interaction between rs699780 and arsenic exposure on T2DM risks. Second, rs699780 is a 3′-UTR SNP located in *NOTCH2*, but there is no current evidence that it is associated with mRNA expression of NOTCH2 based on the gene expression profile database [40]. Thus, additional studies are needed to determine if this genetic variant in *NOTCH2* is associated with differential gene expression that would further validate our finding. Third, the non-monotonically increasing pattern for arsenic exposure and risks of T2DM among C allele carriers of rs699780 may not adequately explain the underlying exposure-response curve. However, the piece-wise regression model assessing the dose-response relationship between arsenic exposure and diabetic risks among the whole population showed that the arsenic exposure in the second quartile (from 2.7 to 20.1 µg/L) was not statistically significant (p-value = 0.07), suggesting that the decreasing trend within this arsenic exposure range may be due to insufficient sample size, unmeasured confounding, or non-differential exposure misclassifications that drive the associations toward the null. Lastly, the diagnosis of T2DM was solely based on hemoglobin A1C levels in follow-up period (2009–2011) in this study. However, hemoglobin A1C levels can be affected by hemolytic anemia and deficiency of micronutrition including vitamin B12 and folate that have been found in Bangladesh population [41]–[43]. Therefore, this study may suffer the caveat of disease misclassifications that will result in potential bias for the association of arsenic exposure with diabetes.

Our study also has several strengths. We utilized continuous arsenic exposure in each participant's primary source of drinking water to assess gene-environment interaction on risks of T2DM. This approach facilitates the understanding how the exposure-response relationship was modified by genetic variants which cannot be ascertained if arsenic exposure was dichotomized. Second, we utilized a hypothesis- driven approach in selecting candidate genes based on their relationship to increased T2DM risk as reported by the DIAGRAM consortium. This consortium combined data from 42,000 controls and 99,000 T2DM cases in an unbiased genome-wide association study to identify novel genetic variants associated with T2DM. Third, inclusions criteria of each SNP in target genes were based on their potential biological effects. Selecting SNPs with possible roles on altering the gene expression or protein function was a more plausible approach compared with inclusions of intronic or intergenic SNPs.

## Conclusions

In summary, we discovered a significant interaction between a 3′-UTR SNP in *NOTCH2* and arsenic exposure from drinking water that increased the risk of T2DM. This finding provides the first human evidence that *NOTCH2* may be a gene potentially involved in arsenic-associated T2DM. We also observed that SNPs located in *ADAMTS9* were nominally associated with an increased risk of T2DM. This finding is consistent with previous association studies discovered in the Caucasian population. Overall, these results indicate that some individuals may be more genetically susceptible to arsenic-induced T2DM.

## Supporting Information

Figure S1 **Scatter plots with arsenic exposure in drinking water versus glycated hemoglobin A1c levels by** ***NOTCH2*** **(rs699780) genotype.** Restricted scatter plots were based on people exposed to water arsenic below 148 μg/L. Sample sizes were 316 for TT carriers and 325 for TC/CC carriers of rs699780. Water arsenic was natural logarithm-transformed.
(TIFF)

Figure S2 **Scatter plots with arsenic exposure in drinking water versus glycated hemoglobin A1c levels by self-reported diabetes.** Restricted scatter plots were based on people exposed to water arsenic below 148 μg/L. Sample sizes were 849 for participants without self-reported diabetes and 46 for those with self-reported diabetes. Water arsenic was natural logarithm-transformed.
(TIFF)

Figure S3 **Exposure-response relationship between arsenic in drinking water and % change of hemoglobin A1c levels by** ***NOTCH2*** **.** Restricted analyses were performed among people exposed to water arsenic below 148 μg/L adjusting for age, sex, BMI, smoking, skin lesion, and arsenic in drinking water using cubic polynomial models. P for interaction was 0.044. Sample sizes were 315 for TT carriers and 324 for TC/CC carriers of rs699780. The solid line indicated the estimated odds ratio using lowest exposure levels of water arsenic as the reference. Water arsenic was natural logarithm- transformed.
(TIFF)

Table S1 **Characteristics of genotyped SNPs.** Abbreviations: 5UTR, 5′-untranslated region; 3UTR, 3′-untranlated region.
(DOCX)

Table S2 **Associations between SNPs and risks of type 2 diabetes.** Abbreviations: 5UTR, 5′-untranslated region; 3UTR, 3′-untranlated region. ^a^ Models were adjusted for age, sex, BMI, smoking, skin lesion, and arsenic in drinking water using penalized splines. ^b^ Q-values were 0.247 for rs17070905, 0.408 for rs17070967, and 0.247 for rs6766801 using FDR method.
(DOCX)

Table S3 **Interaction between SNPs and arsenic in drinking water on risks of type 2 diabetes^a^.** ^a^ Models were adjusted for age, sex, BMI, smoking, skin lesion, SNPs and arsenic in drinking water using piece-wise regression models. ^b^ q-values were calculated using FDR method for p for interaction among whole population. ^c^ P for interactions were 0.613 for rs1051055 (*CDC123*), 0.048 for rs699780 (*NOTCH2*), and 0.219 for rs2688 (*TCF2*) among people exposed to water arsenic less than 148 μg/L.
(DOCX)

Table S4 **Associations between SNPs and % of glycated hemoglobin A1c levels.** ^a^ Models were adjusted for age, sex, BMI, smoking, skin lesion, and arsenic in drinking water using penalized splines. Change of glycated hemoglobin A1c levels were based on 1 unit increase on log water arsenic. ^b^ Q-values were 0.374 for rs17070905, 0.486 for rs17070967, and 0.374 for rs6766801 using FDR method.
(DOCX)

Table S5 **Interaction between SNPs and arsenic in drinking water on % of glycated hemoglobin A1c levels.** ^a^ Models were adjusted for age, sex, BMI, smoking, skin lesion, SNPs and arsenic in drinking water using piece-wise regression models. ^b^ Q-values were calculated using FDR method for p for interaction among whole population.
(DOCX)
