# Supplementary figures and images for: Genetic Susceptible Locus in NOTCH2 Interacts with Arsenic in Drinking Water on Risk of Type 2 Diabetes
Source: PLoS One. 2013 Aug 14;8(8):e70792. doi: 10.1371/journal.pone.0070792 (PMC3743824; doi:10.1371/journal.pone.0070792)

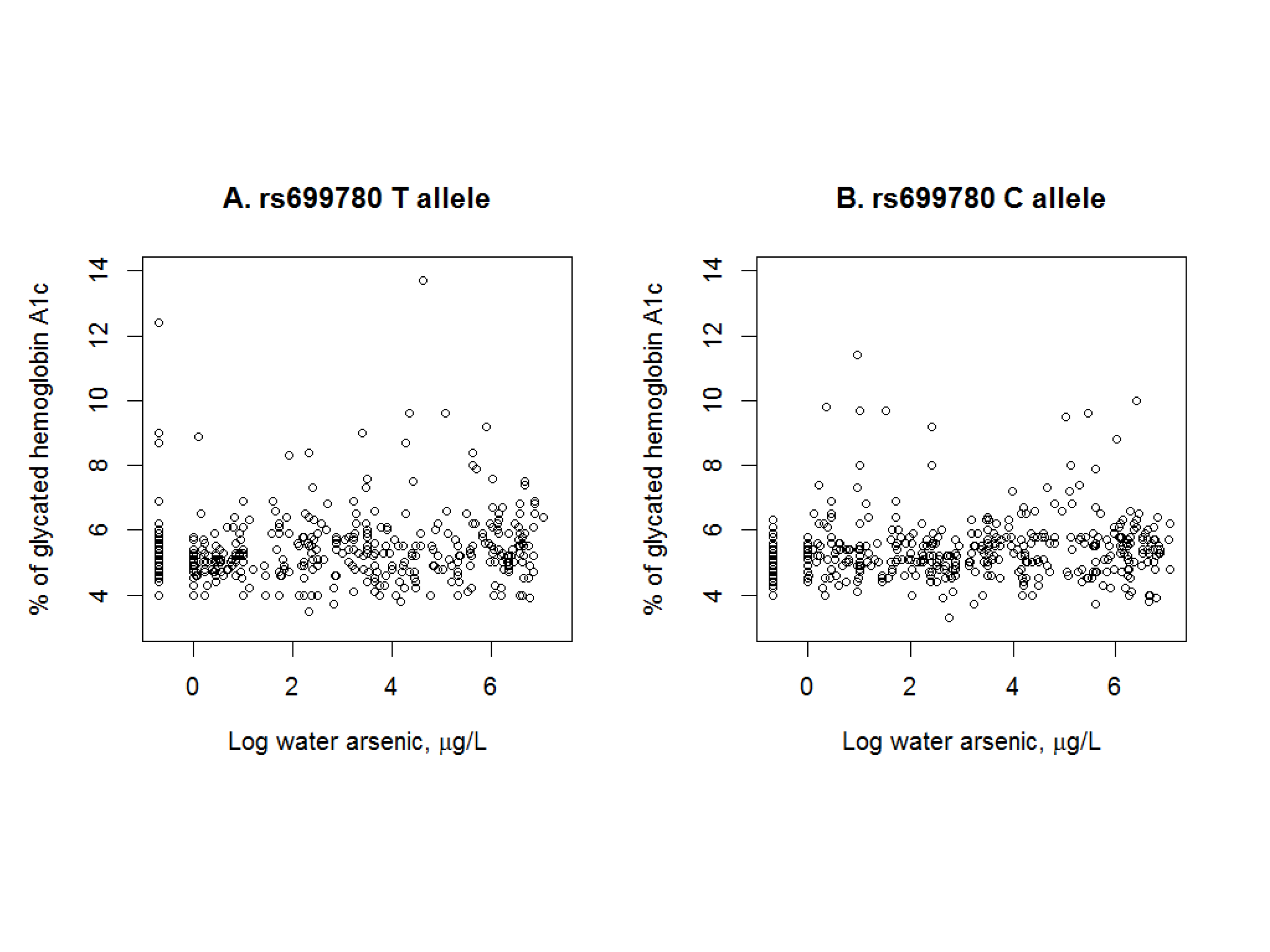

Supplement: Figure S1 — Scatter plots with arsenic exposure in drinking water versus glycated hemoglobin A1c levels by NOTCH2 (rs699780) genotype. Restricted scatter plots were based on people exposed to water arsenic below 148 μg/L. Sample sizes were 316 for TT carriers and 325 for TC/CC carriers of rs699780. Water arsenic was natural logarithm-transformed. (TIFF) [file pone.0070792.s001.tiff]

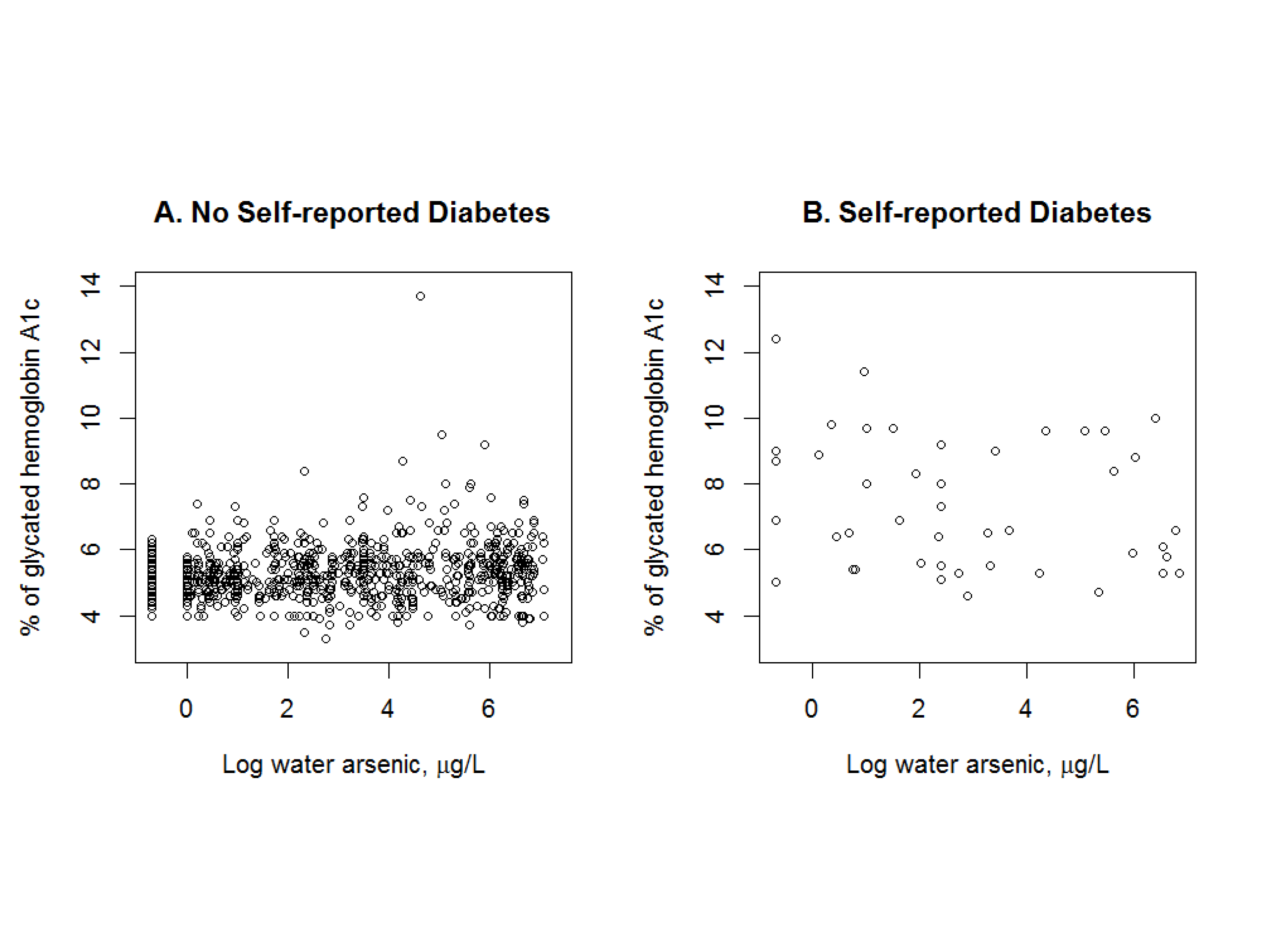

Supplement: Figure S2 — Scatter plots with arsenic exposure in drinking water versus glycated hemoglobin A1c levels by self-reported diabetes. Restricted scatter plots were based on people exposed to water arsenic below 148 μg/L. Sample sizes were 849 for participants without self-reported diabetes and 46 for those with self-reported diabetes. Water arsenic was natural logarithm-transformed. (TIFF) [file pone.0070792.s002.tiff]

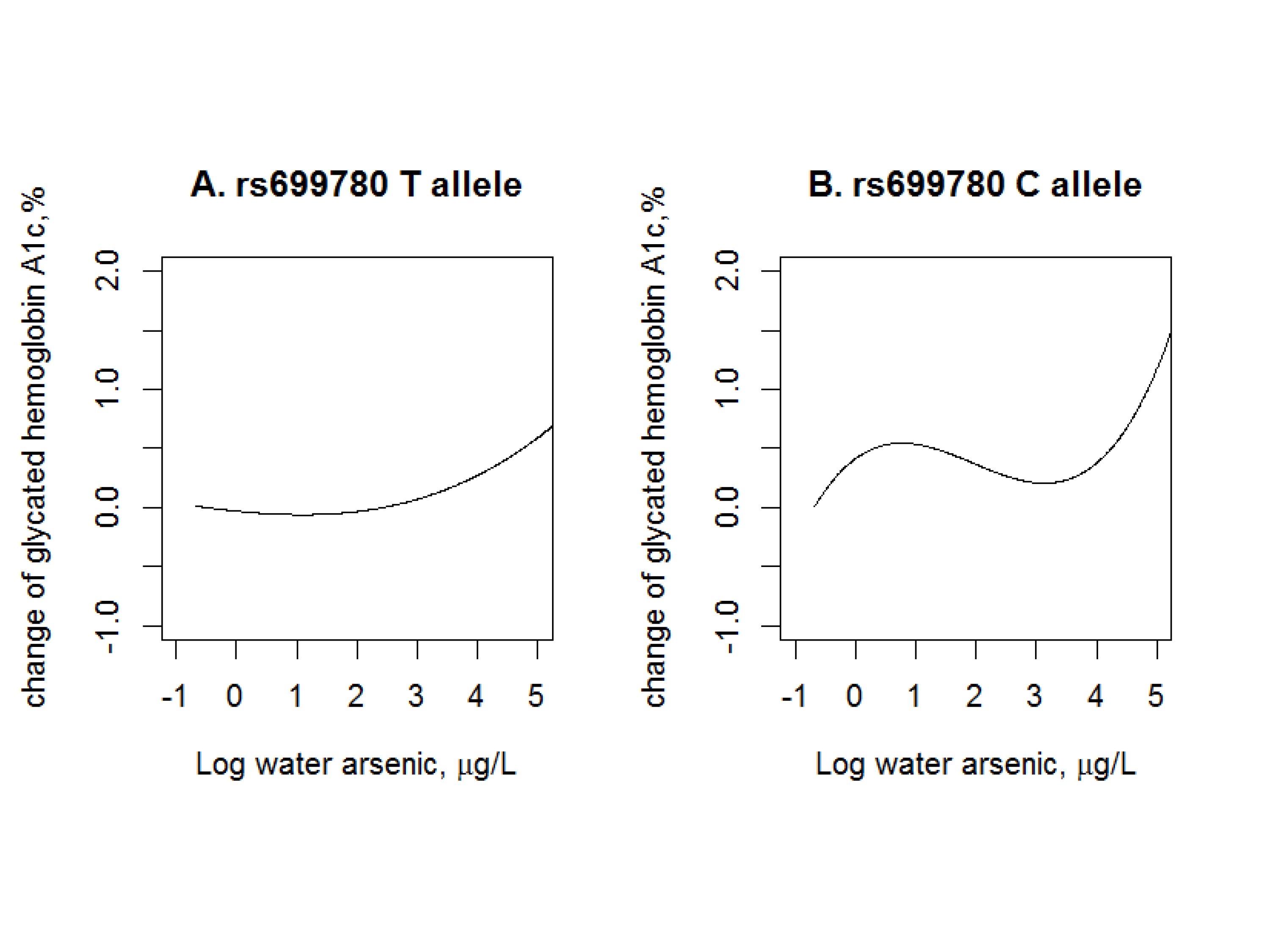

Supplement: Figure S3 — Exposure-response relationship between arsenic in drinking water and % change of hemoglobin A1c levels by NOTCH2 . Restricted analyses were performed among people exposed to water arsenic below 148 μg/L adjusting for age, sex, BMI, smoking, skin lesion, and arsenic in drinking water using cubic polynomial models. P for interaction was 0.044. Sample sizes were 315 for TT carriers and 324 for TC/CC carriers of rs699780. The solid line indicated the estimated odds ratio using lowest exposure levels of water arsenic as the reference. Water arsenic was natural logarithm- transformed. (TIFF) [file pone.0070792.s003.tiff]
