# Supplementary material for: Genetic Susceptible Locus in NOTCH2 Interacts with Arsenic in Drinking Water on Risk of Type 2 Diabetes
Source: PLoS One. 2013 Aug 14;8(8):e70792. doi: 10.1371/journal.pone.0070792 (PMC3743824; doi:10.1371/journal.pone.0070792)
Supplement: Table S1 — Characteristics of genotyped SNPs. Abbreviations: 5UTR, 5′-untranslated region; 3UTR, 3′-untranlated region. (DOCX) [file pone.0070792.s004.docx]

Table S1. Characteristics of genotyped SNPs

| Marker | Gene | Type of SNP | Major Allele | Minor Allele | Minor Allele Frequency |
| --- | --- | --- | --- | --- | --- |
| rs17070905 | ADAMTS9 | 3UTR | G | A | 0.05 |
| rs17070967 | ADAMTS9 | non-synonymous | A | G | 0.05 |
| rs6766801 | ADAMTS9 | 3UTR | A | G | 0.05 |
| rs2058703 | BCL11A | 3UTR | T | C | 0.40 |
| rs1051055 | CDC123 | 3UTR | G | A | 0.27 |
| rs12126 | CDC123 | 3UTR | T | A | 0.11 |
| rs3088440 | CDKN2A | 3UTR | G | A | 0.17 |
| rs1063192 | CDKN2B | 3UTR | T | C | 0.24 |
| rs3217986 | CDKN2B | 3UTR | A | C | 0.17 |
| rs3217992 | CDKN2B | 3UTR | G | A | 0.41 |
| rs11603334 | CENTD2 | 5UTR | C | T | 0.19 |
| rs4646954 | IDE | 5UTR | C | T | 0.15 |
| rs1057128 | KCNQ1 | non-synonymous | G | A | 0.17 |
| rs10798 | KCNQ1 | 3UTR | G | A | 0.45 |
| rs8234 | KCNQ1 | 3UTR | G | A | 0.45 |
| rs343092 | KMGA2 | 3UTR | T | G | 0.43 |
| rs17109924 | LGR5 | non-synonymous | T | C | 0.10 |
| rs1043964 | NOTCH2 | 3UTR | A | G | 0.12 |
| rs699779 | NOTCH2 | 3UTR | T | C | 0.18 |
| rs699780 | NOTCH2 | 3UTR | T | C | 0.30 |
| rs7527186 | NOTCH2 | 3UTR | G | A | 0.11 |
| rs835575 | NOTCH2 | 3UTR | C | A | 0.18 |
| rs835576 | NOTCH2 | 3UTR | A | G | 0.18 |
| rs12911192 | PRC1 | non-synonymous | A | G | 0.17 |
| rs14280 | PRC1 | 3UTR | A | G | 0.08 |
| rs7601 | PRC1 | 3UTR | C | T | 0.43 |
| rs10282940 | SLC30A8 | 3UTR | G | A | 0.10 |
| rs11558471 | SLC30A8 | 3UTR | A | G | 0.26 |
| rs2466293 | SLC30A8 | 3UTR | A | G | 0.42 |
| rs1058166 | TCF2 | 3UTR | A | G | 0.03 |
| RS10962 | TCF2 | 3UTR | C | G | 0.22 |
| rs2688 | TCF2 | 3UTR | A | C | 0.44 |
| rs1549723 | THADA | 3UTR | C | A | 0.25 |
| rs17031056 | THADA | non-synonymous | G | A | 0.27 |
| rs1051334 | TSPAN8 | non-synonymous | T | G | 0.25 |
| rs1801208 | WFS1 | non-synonymous | G | A | 0.09 |
| rs1801212 | WFS1 | non-synonymous | A | G | 0.10 |
| rs734312 | WFS1 | non-synonymous | A | G | 0.40 |

Abbreviations: 5UTR, 5’-untranslated region; 3UTR, 3’-untranlated region.
