# Supplementary material for: Genetic Susceptible Locus in NOTCH2 Interacts with Arsenic in Drinking Water on Risk of Type 2 Diabetes
Source: PLoS One. 2013 Aug 14;8(8):e70792. doi: 10.1371/journal.pone.0070792 (PMC3743824; doi:10.1371/journal.pone.0070792)
Supplement: Table S2 — Associations between SNPs and risks of type 2 diabetes. Abbreviations: 5UTR, 5′-untranslated region; 3UTR, 3′-untranlated region. a Models were adjusted for age, sex, BMI, smoking, skin lesion, and arsenic in drinking water using penalized splines. b Q-values were 0.247 for rs17070905, 0.408 for rs17070967, and 0.247 for rs6766801 using FDR method. (DOCX) [file pone.0070792.s005.docx]

Table S2. Associations between SNPs and risks of type 2 diabetes

|  |  |  |  |  | 95% Confidence Interval | |
| --- | --- | --- | --- | --- | --- | --- |
| Marker | Gene | Type of SNPs | Adjusted Odds Ratio^a^ | P-value | Lower bound | Upper bound |
| rs17070905 | ADAMTS9 | 3UTR | 2.30 | 0.015^b^ | 1.17 | 4.50 |
| rs17070967 | ADAMTS9 | 3UTR | 2.02 | 0.049^b^ | 1.00 | 4.06 |
| rs6766801 | ADAMTS9 | 3UTR | 2.33 | 0.015^b^ | 1.18 | 4.60 |
| rs2058703 | BCL11A | non-synonymous | 1.11 | 0.559 | 0.77 | 1.60 |
| rs1051055 | CDC123 | non-synonymous | 1.00 | 0.998 | 0.67 | 1.49 |
| rs12126 | CDC123 | 3UTR | 0.68 | 0.379 | 0.28 | 1.62 |
| rs3088440 | CDKN2A | 3UTR | 1.07 | 0.787 | 0.67 | 1.68 |
| rs1063192 | CDKN2B | 3UTR | 0.65 | 0.080 | 0.41 | 1.05 |
| rs3217986 | CDKN2B | 3UTR | 0.78 | 0.469 | 0.40 | 1.53 |
| rs3217992 | CDKN2B | 3UTR | 1.09 | 0.648 | 0.76 | 1.54 |
| rs11603334 | CENTD2 | 5UTR | 1.68 | 0.077 | 0.95 | 2.98 |
| rs4646954 | IDE | 3UTR | 1.54 | 0.201 | 0.80 | 2.97 |
| rs1057128 | KCNQ1 | non-synonymous | 1.10 | 0.683 | 0.70 | 1.73 |
| rs10798 | KCNQ1 | 3UTR | 0.86 | 0.564 | 0.51 | 1.44 |
| rs8234 | KCNQ1 | 3UTR | 0.81 | 0.248 | 0.56 | 1.16 |
| rs343092 | KMGA2 | non-synonymous | 0.93 | 0.703 | 0.65 | 1.34 |
| rs17109924 | LGR5 | 3UTR | 1.36 | 0.296 | 0.77 | 2.40 |
| rs1043964 | NOTCH2 | non-synonymous | 0.86 | 0.704 | 0.39 | 1.90 |
| rs699779 | NOTCH2 | non-synonymous | 0.79 | 0.463 | 0.42 | 1.49 |
| rs699780 | NOTCH2 | non-synonymous | 0.68 | 0.066 | 0.44 | 1.03 |
| rs7527186 | NOTCH2 | non-synonymous | 0.77 | 0.378 | 0.43 | 1.38 |
| rs835575 | NOTCH2 | 3UTR | 0.67 | 0.130 | 0.40 | 1.13 |
| rs835576 | NOTCH2 | 3UTR | 0.70 | 0.166 | 0.42 | 1.16 |
| rs12911192 | PRC1 | 3UTR | 0.99 | 0.972 | 0.62 | 1.59 |
| rs14280 | PRC1 | 3UTR | 0.89 | 0.738 | 0.46 | 1.74 |
| rs7601 | PRC1 | 3UTR | 0.85 | 0.380 | 0.59 | 1.22 |
| rs10282940 | SLC30A8 | 3UTR | 1.15 | 0.651 | 0.64 | 2.06 |
| rs11558471 | SLC30A8 | 3UTR | 1.16 | 0.615 | 0.66 | 2.03 |
| rs2466293 | SLC30A8 | 5UTR | 1.37 | 0.088 | 0.95 | 1.97 |
| rs1058166 | TCF2 | 3UTR | 1.76 | 0.233 | 0.70 | 4.44 |
| rs1058166 | TCF2 | 3UTR | 1.76 | 0.233 | 0.44 | 1.10 |
| rs1058166 | TCF2 | 3UTR | 1.76 | 0.233 | 0.66 | 1.33 |
| rs2688 | TCF2 | non-synonymous | 0.94 | 0.718 | 0.65 | 1.46 |
| rs1549723 | THADA | 3UTR | 0.97 | 0.893 | 0.68 | 1.55 |
| rs17031056 | THADA | 3UTR | 1.03 | 0.902 | 0.85 | 1.89 |
| rs1801208 | WFS1 | 3UTR | 0.73 | 0.377 | 0.37 | 1.46 |
| rs1801212 | WFS1 | 3UTR | 0.98 | 0.936 | 0.55 | 1.75 |
| rs734312 | WFS1 | 3UTR | 0.88 | 0.491 | 0.61 | 1.26 |

Abbreviations: 5UTR, 5’-untranslated region; 3UTR, 3’-untranlated region.

^a^ Models were adjusted for age, sex, BMI, smoking, skin lesion, and arsenic in drinking water using penalized splines.

^b^ Q-values were 0.247 for rs17070905, 0.408 for rs17070967, and 0.247 for rs6766801 using FDR method.
