# Supplementary material for: Genetic Susceptible Locus in NOTCH2 Interacts with Arsenic in Drinking Water on Risk of Type 2 Diabetes
Source: PLoS One. 2013 Aug 14;8(8):e70792. doi: 10.1371/journal.pone.0070792 (PMC3743824; doi:10.1371/journal.pone.0070792)
Supplement: Table S3 — Interaction between SNPs and arsenic in drinking water on risks of type 2 diabetesa. a Models were adjusted for age, sex, BMI, smoking, skin lesion, SNPs and arsenic in drinking water using piece-wise regression models. b q-values were calculated using FDR method for p for interaction among whole population. c P for interactions were 0.613 for rs1051055 (CDC123), 0.048 for rs699780 (NOTCH2), and 0.219 for rs2688 (TCF2) among people exposed to water arsenic less than 148 μg/L. (DOCX) [file pone.0070792.s006.docx]

Table S3. Interaction between SNPs and arsenic in drinking water on risks of type 2 diabetes^a^

| Marker | Gene | p for interaction among whole study population | q-value^b^ |
| --- | --- | --- | --- |
| rs17070905 | ADAMTS9 | 0.076 | 0.063 |
| rs17070967 | ADAMTS9 | 0.096 | 0.063 |
| rs6766801 | ADAMTS9 | 0.113 | 0.063 |
| rs2058703 | BCL11A | 0.101 | 0.063 |
| rs1051055 | CDC123 | 0.008^c^ | 0.033 |
| rs12126 | CDC123 | 0.017 | 0.052 |
| rs3088440 | CDKN2A | 0.088 | 0.063 |
| rs1063192 | CDKN2B | 0.138 | 0.067 |
| rs3217986 | CDKN2B | 0.078 | 0.063 |
| rs3217992 | CDKN2B | 0.938 | 0.319 |
| rs11603334 | CENTD2 | 0.024 | 0.061 |
| rs4646954 | IDE | 0.136 | 0.067 |
| rs1057128 | KCNQ1 | 0.107 | 0.063 |
| rs10798 | KCNQ1 | 0.093 | 0.063 |
| rs8234 | KCNQ1 | 0.144 | 0.067 |
| rs343092 | KMGA2 | 0.123 | 0.065 |
| rs17109924 | LGR5 | 0.090 | 0.063 |
| rs1043964 | NOTCH2 | 0.438 | 0.168 |
| rs699779 | NOTCH2 | 0.096 | 0.063 |
| rs699780 | NOTCH2 | 0.003^c^ | 0.021 |
| rs7527186 | NOTCH2 | 0.059 | 0.063 |
| rs835575 | NOTCH2 | 0.115 | 0.063 |
| rs835576 | NOTCH2 | 0.044 | 0.063 |
| rs12911192 | PRC1 | 0.243 | 0.109 |
| rs14280 | PRC1 | 0.960 | 0.319 |
| rs7601 | PRC1 | 0.465 | 0.168 |
| rs10282940 | SLC30A8 | 0.348 | 0.146 |
| rs11558471 | SLC30A8 | 0.553 | 0.194 |
| rs2466293 | SLC30A8 | 0.317 | 0.138 |
| rs1058166 | TCF2 | 0.454 | 0.168 |
| rs10962 | TCF2 | 0.115 | 0.063 |
| rs2688 | TCF2 | 0.003^c^ | 0.021 |
| rs1549723 | THADA | 0.046 | 0.063 |
| rs17031056 | THADA | 0.384 | 0.156 |
| rs1051334 | TSPAN8 | 0.052 | 0.063 |
| rs1801208 | WFS1 | 0.111 | 0.063 |
| rs1801212 | WFS1 | 0.110 | 0.063 |
| rs734312 | WFS1 | 0.449 | 0.168 |

^a^ Models were adjusted for age, sex, BMI, smoking, skin lesion, SNPs and arsenic in drinking water using piece-wise regression models.

^b^ q-values were calculated using FDR method for p for interaction among whole population.

^c^ P for interactions were 0.613 for rs1051055 (*CDC123*), 0.048 for rs699780 (*NOTCH2*), and 0.219 for rs2688 (*TCF2*) among people exposed to water arsenic less than 148 μg/L.
