# Supplementary material for: Genetic Susceptible Locus in NOTCH2 Interacts with Arsenic in Drinking Water on Risk of Type 2 Diabetes
Source: PLoS One. 2013 Aug 14;8(8):e70792. doi: 10.1371/journal.pone.0070792 (PMC3743824; doi:10.1371/journal.pone.0070792)
Supplement: Table S4 — Associations between SNPs and % of glycated hemoglobin A1c levels. a Models were adjusted for age, sex, BMI, smoking, skin lesion, and arsenic in drinking water using penalized splines. Change of glycated hemoglobin A1c levels were based on 1 unit increase on log water arsenic. b Q-values were 0.374 for rs17070905, 0.486 for rs17070967, and 0.374 for rs6766801 using FDR method. (DOCX) [file pone.0070792.s007.docx]

Table S4. Associations between SNPs and % of glycated hemoglobin A1c levels

|  |  |  |  | 95% Confidence Interval | |
| --- | --- | --- | --- | --- | --- |
| Marker | Gene | Adjusted change of glycated hemoglobin A1c, %^a^ | P-value | Lower bound | Upper bound |
| rs17070905 | ADAMTS9 | 0.19 | 0.089^b^ | 0.97 | 1.51 |
| rs17070967 | ADAMTS9 | 0.15 | 0.179^b^ | 0.93 | 1.45 |
| rs6766801 | ADAMTS9 | 0.20 | 0.069^b^ | 0.98 | 1.51 |
| rs2058703 | BCL11A | 0.01 | 0.859 | 0.91 | 1.11 |
| rs1051055 | CDC123 | -0.07 | 0.197 | 0.84 | 1.04 |
| rs12126 | CDC123 | -0.18 | 0.061 | 0.69 | 1.01 |
| rs3088440 | CDKN2A | -0.02 | 0.781 | 0.87 | 1.11 |
| rs1063192 | CDKN2B | -0.09 | 0.127 | 0.81 | 1.03 |
| rs3217986 | CDKN2B | -0.07 | 0.378 | 0.80 | 1.09 |
| rs3217992 | CDKN2B | 0.07 | 0.180 | 0.97 | 1.18 |
| rs11603334 | CENTD2 | 0.14 | 0.091 | 0.98 | 1.34 |
| rs4646954 | IDE | 0.07 | 0.399 | 0.91 | 1.28 |
| rs1057128 | KCNQ1 | 0.00 | 0.954 | 0.88 | 1.13 |
| rs10798 | KCNQ1 | -0.02 | 0.744 | 0.86 | 1.11 |
| rs8234 | KCNQ1 | -0.03 | 0.576 | 0.88 | 1.07 |
| rs343092 | KMGA2 | -0.04 | 0.414 | 0.87 | 1.06 |
| rs17109924 | LGR5 | 0.19 | 0.018 | 1.03 | 1.42 |
| rs1043964 | NOTCH2 | -0.09 | 0.422 | 0.75 | 1.13 |
| rs699779 | NOTCH2 | 0.00 | 0.950 | 0.87 | 1.16 |
| rs699780 | NOTCH2 | -0.03 | 0.557 | 0.87 | 1.08 |
| rs7527186 | NOTCH2 | -0.07 | 0.410 | 0.80 | 1.09 |
| rs835575 | NOTCH2 | 0.00 | 0.975 | 0.88 | 1.14 |
| rs835576 | NOTCH2 | 0.02 | 0.791 | 0.90 | 1.15 |
| rs12911192 | PRC1 | -0.06 | 0.391 | 0.83 | 1.07 |
| rs14280 | PRC1 | -0.03 | 0.688 | 0.82 | 1.14 |
| rs7601 | PRC1 | -0.05 | 0.327 | 0.86 | 1.05 |
| rs10282940 | SLC30A8 | 0.04 | 0.625 | 0.88 | 1.23 |
| rs11558471 | SLC30A8 | 0.03 | 0.637 | 0.90 | 1.19 |
| rs2466293 | SLC30A8 | 0.03 | 0.534 | 0.94 | 1.14 |
| rs1058166 | TCF2 | 0.05 | 0.701 | 0.81 | 1.37 |
| rs10962 | TCF2 | -0.04 | 0.441 | 0.85 | 1.07 |
| rs2688 | TCF2 | 0.03 | 0.577 | 0.94 | 1.12 |
| rs1549723 | THADA | -0.10 | 0.080 | 0.82 | 1.01 |
| rs17031056 | THADA | 0.03 | 0.639 | 0.92 | 1.14 |
| rs1051334 | TSPAN8 | 0.03 | 0.563 | 0.93 | 1.15 |
| rs1801208 | WFS1 | -0.11 | 0.225 | 0.76 | 1.07 |
| rs1801212 | WFS1 | -0.09 | 0.244 | 0.79 | 1.06 |
| rs734312 | WFS1 | -0.06 | 0.271 | 0.86 | 1.04 |

^a^ Models were adjusted for age, sex, BMI, smoking, skin lesion, and arsenic in drinking water using penalized splines. Change of glycated hemoglobin A1c levels were based on 1 unit increase on log water arsenic.

^b^ Q-values were 0.374 for rs17070905, 0.486 for rs17070967, and 0.374 for rs6766801 using FDR method.
