# Supplementary material for: Genetic Susceptible Locus in NOTCH2 Interacts with Arsenic in Drinking Water on Risk of Type 2 Diabetes
Source: PLoS One. 2013 Aug 14;8(8):e70792. doi: 10.1371/journal.pone.0070792 (PMC3743824; doi:10.1371/journal.pone.0070792)
Supplement: Table S5 — Interaction between SNPs and arsenic in drinking water on % of glycated hemoglobin A1c levels. a Models were adjusted for age, sex, BMI, smoking, skin lesion, SNPs and arsenic in drinking water using piece-wise regression models. b Q-values were calculated using FDR method for p for interaction among whole population. (DOCX) [file pone.0070792.s008.docx]

Table S5. Interaction between SNPs and arsenic in drinking water on % of glycated hemoglobin A1c levels

| Marker | Gene | p for interaction among whole study population^a^ | q-value^b^ |
| --- | --- | --- | --- |
| rs17070905 | ADAMTS9 | 0.091 | 0.449 |
| rs17070967 | ADAMTS9 | 0.136 | 0.449 |
| rs6766801 | ADAMTS9 | 0.144 | 0.449 |
| rs2058703 | BCL11A | 0.553 | 0.680 |
| rs1051055 | CDC123 | 0.354 | 0.621 |
| rs12126 | CDC123 | 0.786 | 0.745 |
| rs3088440 | CDKN2A | 0.681 | 0.698 |
| rs1063192 | CDKN2B | 0.498 | 0.677 |
| rs3217986 | CDKN2B | 0.931 | 0.793 |
| rs3217992 | CDKN2B | 0.992 | 0.816 |
| rs11603334 | CENTD2 | 0.707 | 0.698 |
| rs4646954 | IDE | 0.417 | 0.621 |
| rs1057128 | KCNQ1 | 0.248 | 0.621 |
| rs10798 | KCNQ1 | 0.086 | 0.449 |
| rs8234 | KCNQ1 | 0.017 | 0.353 |
| rs343092 | KMGA2 | 0.161 | 0.457 |
| rs17109924 | LGR5 | 0.290 | 0.621 |
| rs1043964 | NOTCH2 | 0.326 | 0.621 |
| rs699779 | NOTCH2 | 0.541 | 0.353 |
| rs699780 | NOTCH2 | 0.023 | 0.680 |
| rs7527186 | NOTCH2 | 0.376 | 0.621 |
| rs835575 | NOTCH2 | 0.093 | 0.449 |
| rs835576 | NOTCH2 | 0.078 | 0.449 |
| rs12911192 | PRC1 | 0.571 | 0.680 |
| rs14280 | PRC1 | 0.861 | 0.792 |
| rs7601 | PRC1 | 0.714 | 0.698 |
| rs10282940 | SLC30A8 | 0.905 | 0.793 |
| rs11558471 | SLC30A8 | 0.655 | 0.698 |
| rs2466293 | SLC30A8 | 0.123 | 0.449 |
| rs1058166 | TCF2 | 0.365 | 0.621 |
| rs10962 | TCF2 | 0.645 | 0.698 |
| rs2688 | TCF2 | 0.491 | 0.677 |
| rs1549723 | THADA | 0.385 | 0.621 |
| rs17031056 | THADA | 0.587 | 0.680 |
| rs1051334 | TSPAN8 | 0.137 | 0.449 |
| rs1801208 | WFS1 | 0.411 | 0.621 |
| rs1801212 | WFS1 | 0.351 | 0.621 |
| rs734312 | WFS1 | 0.939 | 0.793 |

^a^ Models were adjusted for age, sex, BMI, smoking, skin lesion, SNPs and arsenic in drinking water using piece-wise regression models.

^b^ Q-values were calculated using FDR method for p for interaction among whole population.
